# Episodic headache with spontaneous hypothermia reveal Shapiro’s syndrome variant with effectiveness of clonidine therapy

**DOI:** 10.1186/s10194-021-01245-3

**Published:** 2021-04-26

**Authors:** Mickael Aubignat, Melissa Tir, Pierre Krystkowiak, Daniela Andriuta

**Affiliations:** 1 Department of Neurology and Center of Expertise in Parkinson’s disease, University Hospital of Amiens, CHU Amiens-Picardie, 80 054 Cedex 1 Amiens, France; 2 Laboratory of Functional Neurosciences (EA 4559), University Hospital of Amiens, Amiens, France

**Keywords:** Headache, Hypothermia, Hyperhidrosis, Shapiro’s syndrome, Parkinson’s disease, Clonidine

## Abstract

**Background:**

Episodic headache with spontaneous hypothermia constitute an uncommon association and is not well recognized in the International Classification of Headache Disorders (ICHD-3). Spontaneous periodic hypothermia, also called Shapiro’s syndrome, is a rare disease characterized by hypothermia attacks associated or not with hyperhidrosis without any triggering factor.

**Case presentation:**

We report a rare case of Shapiro’s syndrome variantrevealed by episodes of headache with spontaneous hypothermia witheffectiveness of clonidine therapy in a 76-year-old Parkinson’s disease woman.

**Conclusions:**

In the literature, apart from Shapiro’s syndrome, headache withhypothermia seem to occur very rarely. In our case,these symptoms may be considered as a very rare non-motor fluctuation ofParkinson’s disease.

## Background

Episodic headache with spontaneous hypothermia constitute an uncommon association and is not well recognized in the International Classification of Headache Disorders (ICHD-3) [1]. Spontaneous periodic hypothermia, also called Shapiro’s syndrome, is a rare disease (less than 60 cases described) characterized by hypothermia attacks associated or not with hyperhidrosis without any triggering factor [2]. First described by William Shapiro in 1969, Shapiro’s syndrome in the strict sense is characterized by the triad of hypothermia, hyperhidrosis, and agenesis of the corpus callosum (ACC) [3]. However, since its description, cases with a similar presentation without ACC have been described, sometimes associated with various neurological pathologies (e.g. multiple sclerosis, brain tumors, autoimmune encephalitis, brain infection, subarachnoid hemorrhages…) [4–7]. Generally, headaches are uncommon and rarely in the foreground in Shapiro’s syndrome [2, 8]. In this paper, we report an unusual case of Shapiro’s syndrome variant revealed by episodes of headache with spontaneous hypothermia in a parkinsonian patient.

## Case presentation

**Fig. 1:**
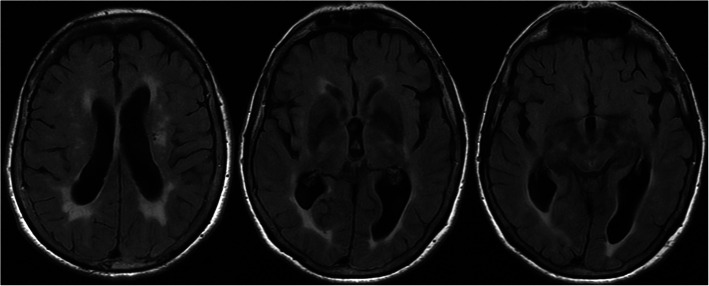
Axial T2-FLAIR brain MRI: left anterior choroid stroke sequelae and significant peri ventricular vascular white matter hyperintensities without other abnormal findings

A 76-year-old woman with a history of Parkinson’s disease (PD) for seventeen years, high blood pressure, atrial fibrillation, and stroke of the left anterior choroidal artery at the age of 68 years was initially referred for acute headache that started in a few minutes and hypothermia of 33.2 °C. She had no headache history. Her medical treatment included amlodipine 5 mg, perindopril 8 mg, fluindione 10 mg, and 1 300 mg per day of a Levodopa Equivalent Dose. The hemodynamic state was stable, but the patient was confused, pale, and sweating. Headache was holocranial, tightness type, not pulsating, moderate without phono-photophobia, nausea, vomiting or meningeal stiffness. There had been no sign of aura. Brain MRI showed left anterior choroid stroke sequelae and significant vascular white matter hyperintensities without other abnormal findings (Fig. 1). EEG showed diffuse slowing without epileptic signals. The EKG was normal, notably without Osborn’s J wave. Various blood tests were normal, as were cerebrospinal fluid tests. Furthermore, no environmental factors of hypothermia were noted. The resulting outcome was spontaneous and favorable, resolving within a few hours with only partial amnesia of the episode. The patient was subsequently rehospitalized three times within two months for similar episodes of acute headache with spontaneous hypothermia between 33 and 34 °C, with hyperhidrosis and confusion (disorientation in time and space and psychomotor slowdown without aphasia, iterative questions or neurological deficit). Each time, headache appear in few minutes without warning signs and without triggering factor. It was always holocranial with a tightness type and moderate intensity. During each hospitalization, clinical examinations including extensive infectious and endocrine studies were normal and the outcome was favorable, resolving within a few hours and sometimes associated with partial episode amnesia.

**Fig. 2:**
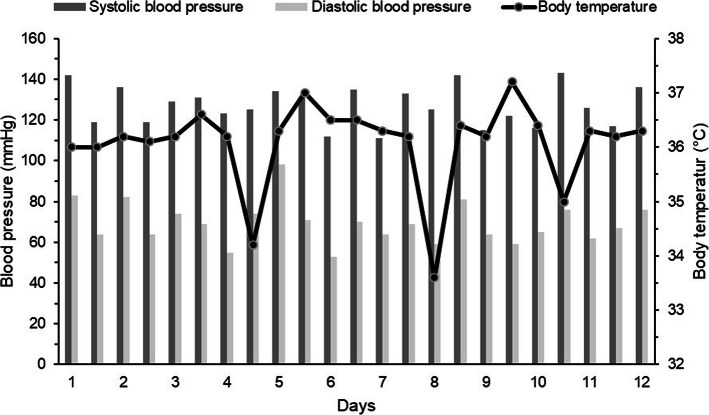
Evolution of blood pressure and body temperature over twelve days with measurement every twelve hours at 6:00 AM and 6:00 PM

After experiencing similar recurring episodes, the patient was hospitalized again. During this hospitalization, four identical episodes were noted, each with a stable hemodynamic state and hypothermia between 32.5 and 34 °C (Fig. 2). The resulting outcome of each episode was spontaneous and favorable, resolving within few hours (between 4 and 12 h). During these episodes, no evidence of motor fluctuations of PD were noted. Assuming non-motor fluctuations (dysautonomic fluctuations) of PD to be the cause, 24-h heart rate and blood pressure monitoring, (123)I-meta-iodobenzylguanidine (MIBG) cardiac scintigraphy, a levodopa challenge, and multiple orthostatic hypotension tests were performed. All exams were normal. Consistent with the hypothesis of Shapiro’s syndrome variant associated with PD, treatment with clonidine 0.15 mg twice daily was started. Following initiation of this treatment, the patient no longer experienced episodes of headache with spontaneous hypothermia over a follow-up of more than four years.

## Discussion

Headache with spontaneous hypothermia are not recognized in the ICHD-3 [1]. Therefore, the headache present in our patient could be classified as a *headache attributed to other non-vascular intracranial disorder* (ICHD-3 7.8) or *headache attributed to other disorder of homoeostasis* (ICHD-3 10.7) or *headache not elsewhere classified* (ICHD-3 14.1). Despite a very exhaustive assessment, no cause could be found to explain these episodes of hypothermia present in our patient [9]. The absence of an etiology to explain these recurrent episodes of hypothermia with hyperhidrosis led us to suspect the diagnosis of Shapiro’s syndrome variant. Little is known about the pathophysiology of Shapiro’s syndrome. It could be secondary to hypothalamic abnormalities not visible on common 1.5 or 3 Tesla brain MRI [2, 8]. Some authors also mention deregulation of neurotransmitters such as dopamine, serotonin, norepinephrine, or melatonin [10, 11]. Others evoke a diencephalic epileptic origin [12]. However, no epileptic signals have been identified and antiepileptic drugs seem to be ineffective in Shapiro’s syndrome. The remarkable effectiveness of clonidine in our patient has also been reported in some cases of Shapiro’s syndrome [2, 8]. The prevalence of headaches would be 40.8 % in the PD population compared to 69.4 % in the general population and only 5.8 % in Shapiro’s syndrome patients [2, 13]. In the literature, apart from Shapiro’s syndrome, headaches with hypothermia seem to occur very rarely. Only one case of migraines with hypothermic auras is described [14]. However, our patient had no migraine criteria and no headache history [1]. Moreover, spontaneous hypothermia seems to be a rare event in PD, with less than ten cases described in the literature [15]. In most of these cases, a hypothermic episode occurred only once. To our knowledge, only one case similar to ours has been described in a PD patient [16]. In this case, the authors report hypothalamic deposits of alpha-synuclein on postmortem brain examination secondary to PD progression. These deposits, invisible on brain MRI, could cause disorders in hypothalamic thermoregulatory centers and cause the symptomatology of Shapiro’s syndrome. Thus, Shapiro’s syndrome variant in a parkinsonian patient could be secondary to hypothalamic deposits of alpha-synuclein and as such may be considered as a very rare non-motor fluctuation of PD [17]. In conclusion, episodic headache with spontaneous hypothermia appear to be very rare in clinical practice and little is known about its pathophysiology. In this context, Shapiro’s syndrome variant must be mentioned and clonidine can be an effective therapy.

## Data Availability

Data is available with corresponding author upon request.

## Abbreviations

ACC: Agenesis of the corpus callosum
ICHD-3: International Classification of Headache Disorders
MRI: Magnetic Resonance Imagin
PD: Parkinson’s disease
